# Improving the effectiveness of sickness benefit case management through a public-private partnership? A difference-in-difference analysis in eighteen Danish municipalities

**DOI:** 10.1186/s12889-017-4236-5

**Published:** 2017-04-18

**Authors:** Malene Rode Larsen, Birgit Aust, Jan Høgelund

**Affiliations:** 1 The Danish National Centre for Social Research, Herluf Trolles Gade 11, DK-1052 Copenhagen K, Denmark; 2 The National Research Centre for the Working Environment, Lersø Parkallé 105, DK-2100 Copenhagen Ø, Denmark; 3 The Danish Working Environment Authority, Landskronagade 33, DK-2100 Copenhagen Ø, Denmark

**Keywords:** Denmark, Effect evaluation, Hazard rate model, RTW, Sickness benefit duration, Sick leave, Work resumption

## Abstract

**Background:**

The aim of this study was to investigate whether a multidimensional public-private partnership intervention, focussing on improving the quality and efficiency of sickness benefit case management, reduced the sickness benefit duration and the duration until self-support.

**Methods:**

We used a difference-in-difference (DID) design with six intervention municipalities and 12 matched control municipalities in Denmark. The study sample comprised 282,103 sickness benefit spells exceeding four weeks. The intervention group with 110,291 spells received the intervention, and the control group with 171,812 spells received ordinary sickness benefit case management. Using register data, we fitted Cox proportional hazard ratio models, estimating hazard ratios (HR) and confidence intervals (CI).

**Results:**

We found no joint effect of the intervention on the sickness benefit duration (HR 1.02, CI 0.97–1.07) or the duration until self-support (HR 0.99, CI 0.96–1.02). The effect varied among the six municipalities, with sickness benefit HRs ranging from 0.96 (CI 0.93–1.00) to 1.13 (CI 1.08–1.18) and self-support HRs ranging from 0.91 (CI 0.82–1.00) to 1.11 (CI 1.06–1.17).

**Conclusions:**

Compared to receiving ordinary sickness benefit management the intervention had on average no effect on the sickness benefit duration or duration until self-support. However, the effect varied considerably among the six municipalities possibly due to differences in the implementation or the complexity of the intervention.

**Electronic supplementary material:**

The online version of this article (doi:10.1186/s12889-017-4236-5) contains supplementary material, which is available to authorized users.

## Background

Disability is a major human burden and a huge challenge in many countries: it reduces the labour supply and forces society to allocate considerable resources for treatments and cash transfers [1–6]. To reduce the societal burden of work-related disability, decision-makers and researchers search for effective return-to-work (RTW) interventions for sick-listed employees, and numerous studies have evaluated, among others, health care interventions [7–9], community- and workplace-based interventions [7, 10, 11] and RTW coordination programmes [12].

Despite extensive research activities in this area, effects tend to be small [7, 13]. This might be due to the complexity of the RTW process involving a number of stakeholders such as the sick-listed, the employer, the insurance agency, and the healthcare system [14–16] all of which need to work together despite potential differences in their goals and orientations [16–18]. The important role of better coordination between these stakeholders has often been pointed out [1, 19, 20] and the impact of stakeholder coordination on RTW has been tested in a number of studies [21]. In a systematic review and meta-analysis Schandelmaier et al. studied the effectiveness of RTW coordination, conducted either by a RTW-coordinator or a team coordinating services and communication between the stakeholders involved. Based on nine randomized controlled trials (RCT’s) they concluded that there is moderate quality evidence that RTW coordination interventions result in small relative increases in RTW [12].

Some studies have pointed out that implementing interventions with several interactive elements may be subject to reinvention and implementation failures [22–24], potentially reducing intervention effects. For example, a detailed evaluation of the intervention project ‘the Danish RTW-programme’, which among other aspects introduced interdisciplinary RTW teams, showed large variations between municipalities for most implementation aspects [25]. Large variations in the implementation were also found in an intervention focussing on improvements of sickness benefit management for beneficiaries with mental health problems [26]. Among the barriers for implementation were stakeholders’ different interpretations of sickness absence legislation, competing rehabilitation alternatives and lack of managerial support for the intervention, while the motivation and availability of resources to solve disagreement through extensive communication was an important facilitator for the implementation. Overall, the study found delayed RTW compared to ordinary case management [27] and in a comprehensive process evaluation the authors identified a number of implementation failures [28].

In this article we present results from a three-year multidimensional public-private partnership intervention focussing on improving the quality and efficiency of sickness benefit case management in six Danish municipalities. Within the Danish system, municipal social insurance officers (SIOs) are responsible for case management of sickness absence beneficiaries, including coordinating RTW activities with relevant stakeholders, such as employers and health professionals. Municipalities can design and implement their own sickness benefit management policies and procedures within the framework of the sickness benefit act. The intervention, which was provided by the six municipalities in cooperation with the private company Falck Health Care (Falck), consisted of various elements. For example measures that enabled SIOs to work more efficiently (e.g. increased resources and improved tools for documentation and case management) and more structured (e.g. introduction of uniform case management standards). Another element consisted in strengthening the competencies for guiding sick-listed individuals through their long-term sickness absence (e.g. further education courses on a variety of topics in relation to sickness benefit management).

The aim of the intervention was, through the improvement in the quality and efficiency of sickness benefit case management, to support a faster RTW, i.e. to reduce the duration of sickness benefit spells, especially the long-lasting ones (exceeding 52 weeks). To test if the intervention achieved a faster RTW we measured two outcomes: the sickness benefit duration (primary outcome) and the time until self-support (secondary outcome). For both outcomes we also tested if there was a distinct effect for spells exceeding 52 weeks.

## Methods

### Study design

We used a difference-in-difference (DID) design to measure the intervention effect: we compared the before-after development in the sickness benefit duration and time to self-support in six intervention municipalities with the before-after development in 12 control municipalities. The study period consisted of a pre-intervention period and a post-initiation period. The pre-intervention period was about four years in all the intervention municipalities. The post-initiation period lasted from the initiation of the intervention (ranging from August 2008 to September 2009 across the six intervention municipalities) through February 2013 when our study period ended. Consequently, the length of the post-initiation period varied between 42 and 55 months across the municipalities depending on the initiation date. We used similar study periods for the control municipalities.

### Study setting

In Denmark municipalities administer the national sickness benefit act, which covers wage earners, the self-employed and unemployed persons in the unemployment insurance scheme. Over the past decade the sickness benefit scheme has undergone a number of reforms and changes [29], with further changes implemented in 2014. Here we describe the stipulations effective during the study period: sick-listed can receive sickness benefits for up to 52 weeks. The first weeks of sickness benefits for employees are financed by the employer. The duration of this employer financed period has been two weeks until 2008, three weeks until 2012 and has since been four weeks. After the employer financed period sickness benefits are financed by the municipalities and the state. The benefit period can be prolonged under certain conditions, e.g. if vocational rehabilitation may help the sick-listed become fit for work.

The municipal SIO must perform a sickness benefit case management interview with all sick-listed within eight weeks after the first day of sick leave. Thereafter, the municipal SIO must perform an interview every fourth week in complex spells and every eighth week in uncomplicated spells. In connection with the first interview the SIO must develop a RTW plan. Drawing on the interview and on health-related, occupational and social information about the sick-listed, the SIO must assess the sick-listed’s eligibility for continued benefit receipt and support RTW. To promote RTW, the SIO can initiate vocational rehabilitation, e.g. part-time sick leave, education and wage-subsidised employment.

### Sickness benefit management in the intervention municipalities

All sickness absence beneficiaries in the six intervention municipalities received sickness benefit case management provided by the municipalities in cooperation with Falck, one of the biggest Danish private providers of health care services. The agreements between Falck and the municipalities were based on a ‘no cure, no pay’ principle. The agreements specified i) the elements of the intervention, ii) Falck’s minimum resource investment during a three-year contract period, and iii) the way in which the partnership success was to be measured based on a one-year evaluation period, assessing success by various criteria (e.g. reduction in the municipalities’ sickness benefit expenditures). If the success criteria were fulfilled, the economic gain was divided equally between Falck and the municipality; if not, Falck covered the entire costs of the intervention.

As Danish municipalities can make partnership agreements with private companies, participants (i.e. the sickness absence beneficiaries in the six intervention municipalities) were not asked for permission, nor could they refuse to accept the intervention. Falck invested considerable resources in providing additional SIOs, administrative staff and specialists, e.g. psychiatrists, enabling SIOs to speed up the medical diagnosing and treatment. Falck also implemented new administrative tools for documentation and case flow management, e.g. automatic notification about spells needing assessment and discussion, and the number of long-lasting or closed spells for each SIO. New procedures to further improve the administrative workflow and provide uniform case management standards were introduced. In addition, the intervention included further education, e.g. courses on management, better cooperation, the sickness benefit act and case management methods. Furthermore, Falck stressed the importance of increasing the involvement of workplaces in the RTW process, e.g. through increased contact to the employer of the sick-listed. Finally, the municipalities could implement Falck’s rehabilitation model with integrated case management, job coaching and health care services (with diagnoses and treatment by e.g. physiotherapists and psychologists). In this model the SIO, the job coach, and medical expert are located in the same building where they cooperate to establish RTW interventions that better integrate medical and employment oriented aspects.

### Sickness benefit management in the control municipalities

The sickness absence beneficiaries in the 12 control municipalities received ordinary sickness benefit management (OSBM) according to the stipulations in the national sickness benefit act (see ‘Study setting’). However, as Danish municipalities can design and implement their own sickness benefit management policies and procedures under the sickness benefit act, the OSBM may vary. We therefore assessed the control municipalities’ activities aimed at improving their sickness benefit management during the study period (see ‘Data and outcome measures’). Consequently, in this study we investigate whether the intervention had a positive effect over and above the effect of the OSBM which may also have developed during the study period.

### Selection of intervention and control municipalities

The intervention municipalities were municipalities that were approached by Falck and which, following negotiations on a partnership agreement, decided to participate. While eight municipalities decided to participate, two of them established the agreements too recently for inclusion in our study. Therefore, only six intervention municipalities were included in our analyses.

Based on the work of Graversen et al. [30] we selected control municipalities with structural conditions and geographic locations similar to those of the intervention municipalities. Using unique register data for all Danish municipalities, Graversen et al. [30] coded 145 variables for measuring several individual, municipal and regional characteristics that may affect the number of persons receiving sickness benefits exceeding four weeks. The individual characteristics included labour market experience, housing composition, and use of health care services. The municipal and regional characteristics included unemployment rate in the commuting area, number of inhabitants, and composition of job skills among employed people.

Graversen et al. [30] estimated the 2011 average municipal sickness benefit rate, i.e. the average number of sickness benefit weeks divided by 52 weeks, as could be expected given the characteristics of each municipality and its inhabitants. For each intervention municipality we selected two control municipalities from the same commuting area and with a similar expected sickness benefit rate. Because the local unemployment rate may affect the chances of RTW [31], we used a geographic criterion to ensure that sickness absence beneficiaries in the intervention and the control municipalities had similar employment opportunities. Additional file 1: Table S1 shows the predicted average sickness benefit rates in 2011 in the intervention and control municipalities, respectively.

The population size (2014) of the six intervention municipalities (Assens, Herning, Hjørring, Holbæk, Horsens and Kolding) ranged from 41,037 in Assens (the 55th largest of the 98 Danish municipalities) to 90,066 in Kolding (the 10th largest) with an average of approx. 73,000 inhabitants. The population size of the 12 control municipalities ranged from 26,989 in Lejre (the 79th largest) to 109,652 in Vejle (the 6th largest) with an average of approx. 47,700 inhabitants.

### Data and outcome measures

This study was based on data from Danish national administrative registers all of which we accessed through Statistics Denmark after the study had been registered at the Danish Data Protection Agency (https://www.datatilsynet.dk/). We used the ‘Danish Register for Evaluation of Marginalisation’ (DREAM) [32], containing weekly individual-level recordings of all social transfer payments of all Danish citizens from January 2005 through February 2013 (see ‘Results’ for the number of sickness benefit spells in the study population), and other registers containing individual-level data on a broad range of socio-economic characteristics. The different registers were linked through anonymised personal identification codes from the Central Population Register, which assigns a unique number to each resident in Denmark. According to Danish law informed consent from participants is not required for using these data.

We calculated our primary outcome measure, the sickness benefit duration, as the number of consecutive weeks of receipt of sickness benefits until the first week without sickness benefits.^1^ Our population comprised all sickness benefit spells exceeding four weeks (see ‘Results’). We calculated our secondary outcome measure, the time to self-support, as the number of consecutive weeks with sickness benefit receipt followed by another cash transfer benefit, e.g. unemployment benefits or social assistance, until no social transfer payments were received. Similar to a study by Nielsen et al. [33], we define self-support as being economically self-supporting, which is equivalent to receiving no social transfer payments other than payments related to education. Spells that ended, because our study period ended or the beneficiary died or moved to another municipality, were censored.

To assess the extent to which the control municipalities were engaged in additional activities or projects aimed at improving their sickness benefit management during the study period, we conducted telephone interviews with a representative from each of the 12 control municipalities, predominantly the department manager (see 'Results').

### Coding of intervention variables

In a DID design the coefficient of the interaction term between the intervention-control group dummy variable and the before-after intervention dummy variable depicts the intervention effect ([34]:233).

In our analyses the intervention variable had the value 1 for individuals in the six intervention municipalities and 0 for individuals in the 12 control municipalities.

In each intervention municipality the before-after dummy variable equalled 0 in sickness benefit cases starting and ending before the intervention started and equalled 1 in cases starting after the intervention started. In sickness benefit cases starting before the intervention started and ending after the intervention started the before-after dummy variable equalled 0 until the intervention started and then the variable changed to 1.

In the two control municipalities that were matched to an intervention municipality, we coded the before-after dummy variable in the same way as in the intervention municipality. Consequently, for sickness benefit spells with identical starting and ending dates the before-after dummy variable had, at all benefit durations, the same value for individuals from the intervention municipality and individuals from the two control municipalities. With this coding we made sure that variation across the six intervention municipalities in the initiation dates of the intervention did not bias the estimated treatment effect.

### Baseline variables

Our baseline variables were gender, age, citizenship, months of schooling, marital status, pre-school children, previous sick-listing, number and length of hospitalizations, and number of contacts with general practitioners (GPs), medical specialists, physiotherapists and psychologists. Previous sick-listing was measured as the number of weeks sick-listed during the 52 weeks preceding the first week of each sickness benefit spell. All other baseline variables were measured either in the calendar year preceding the sickness benefit spell or on January 1 of the same calendar year as the first day of the sickness benefit spell.

### Data analysis

Our analyses were performed with STATA version 13. We tested group differences using χ^2^tests for categorical variables and *t-*tests for continuous variables. To test differences in the sickness benefit duration and time to self-support between individuals in the intervention and the control municipalities, we fitted Cox proportional hazard ratio models using Stata’s stcox procedure. All models included baseline variables entered as time-invariant control variables. We report hazard ratios (HR) and 95% confidence intervals (CI) for the estimated intervention effects. To test whether the intervention had a specific effect on reducing long-lasting spells, we allowed the HR to change when the duration exceeded 52 weeks.^2^ We performed this test using the time varying covariate (tvc) option in Stata’s stcox procedure. We estimated an intervention effect jointly for the six intervention municipalities and separately for each of the six intervention municipalities. As standard errors (SEs) might be clustered within municipalities, we used Stata’s robust-clustered SE option for the joint analysis of the six intervention municipalities.^3^

### Robustness analyses

To assess the robustness of our findings, we performed four analyses. First, we indirectly tested the DID before-after design’s ‘common-trends assumption’, i.e. that the development in the outcome during the post-initiation period in the intervention group would be similar to the outcome development during the post-initiation period in the control group had there been no intervention ([34]:230). Directly testing this assumption is impossible, because we cannot observe how the post-initiation development of individuals in the intervention group would have been without the treatment. Instead, we tested whether the pre-intervention development in the outcome in the intervention and control group differed jointly. We performed a similar test for each of the separate intervention municipalities and its two corresponding control municipalities. As we performed these tests in a Cox proportional hazard ratio model including dummy variables for each year in the study period, we were able to test for intervention-control group differences in each of the four pre-intervention years.

Second, we tested the robustness of the estimated intervention effects by re-running the estimated hazard models with only one control municipality for each intervention municipality, i.e. we removed the most deviant control municipality in terms of the predicted average municipal sickness benefit rate in 2011 (see ‘Selection of intervention and control municipalities’). We conducted this robustness analysis for the six intervention municipalities both jointly and separately.

Third, we applied a Bonferroni-correction to the significance level of the results of the separate analyses of each of the six intervention municipalities and its two corresponding control municipalities. Since we are testing several models the likelihood of false positive test results increases (i.e. the likelihood of making a type I error increases). The Bonferroni-correction adjusts for this by reducing the significance level from α to α/n. Applied to our study the Bonferroni-corrected significance threshold for the separate analyses becomes 0.05/6 = 0.0083.

Fourth, we tested whether three of the control municipalities’ participation in ‘the Danish RTW programme’ affected the estimated effect of the intervention, i.e. we removed the three municipalities from the estimation of the separately estimated municipal effects.

## Results

Telephone interviews with a representative from each of the 12 control municipalities showed that all control municipalities were engaged in one or more activities during the study period that may have affected their OSBM. Seven control municipalities participated in externally run research projects aimed at returning (in some cases quite specific) groups of sickness absence beneficiaries to work, e.g. through improved sickness benefit case management. These projects comprised relatively small samples of sickness absence beneficiaries. Seven control municipalities hired external consultants to improve their documentation and workflow procedures, e.g. through lean processes, and five control municipalities injected substantial municipal financed resources into the sickness benefit department to reduce SIO caseloads. Five municipalities established internal projects aimed, e.g. at changing the workflow in the sickness benefit department.

The extent to which the 12 control municipalities were engaged in the aforementioned activities varied. However, most did not participate in a sickness benefit case management intervention of a similar scope in terms of inclusion of sickness absence beneficiaries, length, intensity and range of elements as the intervention in the six intervention municipalities. The research project ‘the Danish RTW programme’ [33, 35, 36], in which three control municipalities participated, represents the single activity resembling or surpassing the present intervention in scale and intensity. In sum, our data indicate that OSBM varies between municipalities. This finding is in line with previous studies of OSBM in Danish municipalities [37, 38]. Our findings also suggest that most municipalities constantly try to develop their OSBM. In effect this means that we study whether the Falck intervention had a positive effect over and above the effect of a more or less continuously developing OSBM.

The central elements of the intervention and the extent to which they were implemented in each intervention municipality are shown in Table 1. While some elements were implemented in all six intervention municipalities, others were implemented only in some, e.g. because the municipality already had similar services or procedures [39]. Only one of the intervention municipalities, Horsens, implemented all of the central elements.

**Table 1:** Implementation of central elements of the intervention

Intervention element	Assens	Herning	Hjørring	Holbæk	Horsens	Kolding
Additional resources, e.g. additional SIOs	+	+	+	+	+	+
Additional health care services	+	(+)	+	(+)	+	(+)
New administrative tools for documentation and management of case flow	+	+	+	+	+	+
New workflow procedures and quality standards for case management	+	(+)	+	+	+	+
Development of vocational rehabilitation measures	+	0	+	0	+	(+)
Implementation of Falck’s rehabilitation model with integrated case management	0	0	(+)	0	+	0
Further education of staff	+	(+)	+	(+)	+	+

Source: Bille et al., 2013 [39]

+: Fully implemented, (+): Partly implemented, 0: Not implemented

Our study population comprised 282,103 sickness benefit spells exceeding four weeks during the study period; 110,291 spells in the six intervention municipalities and 171,812 spells in the 12 control municipalities, see Table 2. Thus, the average number of spells was larger in the intervention municipalities (approx. 18,400) compared to the control municipalities (approx. 14,300) with considerable variation between municipalities. These differences largely reflect differences in the population size of the municipalities: the average number of inhabitants in the intervention municipalities exceeds that of the control municipalities (see ‘Selection of intervention and control municipalities’). However, in both the intervention and control municipalities, the spells had a mean and a median duration of 22 and 12 weeks, respectively.

**Table 2:** Number of sickness benefit spells in the intervention and control municipalities during the study period

Intervention municipalities	Number of sickness benefit spells	Control municipalities	Number of sickness benefit spells
Assens	13,091	Nordfyns	9,972
		Nyborg	10,298
Herning	23,902	Ringkøbing-Skjern	16,399
		Ikast-Brande	12,058
Hjørring	2,354	Jammerbugt	13,098
		Brønderslev	10,930
Holbæk	20,798	Sorø	9,399
		Lejre	7,606
Horsens	24,732	Favrskov	13,375
		Silkeborg	26,131
Kolding	25,414	Middelfart	11,655
		Vejle	30,891
Total	110,291	Total	171,812

Baseline descriptive statistics for individuals in the intervention and control municipalities, respectively, are shown in Table 3. Standardized Mean Differences (SMDs) show no sign of statistical differences between the intervention and control municipalities on any of the baseline variables.

**Table 3:** Baseline descriptive statistics for sickness absence beneficiaries in the intervention and control municipalities

	Intervention (*N* = 13,617)		Control (*N* = 21,748)		
	Percent		Percent		Standardized Mean Difference (SMD)^a^
Binary variables					
Gender (ref = male)	56.4		57.0		0.009
Citizenship (ref = non-Western country)					
Danish	95.9		96.6		0.026
Western country (excl. Danish)	1.4		1.6		0.012
Marital status (ref = not single)	28.2		25.6		0.041
Pre-school children (ref = no)	18.1		18.8		0.013
	Mean	(SD)	Mean	(SD)	
Continuous variables					
Age	41.9	11.5	42.6	11.4	0.041
Months of schooling	148.7	31.0	149.5	30.8	0.020
Weeks sick-listed the preceding 52 weeks	3.6	7.6	3.8	7.8	0.016
Number of visits to GP	9.5	8.7	9.2	8.7	0.030
Number of visits to medical specialists	0.9	2.8	0.8	2.3	0.024
Number of visits to physiotherapists	1.9	5.9	2.3	6.9	0.047
Number of visits to psychologists	0.1	1.1	0.1	0.9	0.015
Number of hospitalisations	0.2	0.6	0.2	0.8	0.011
Length of hospitalisation (no. of bed days)	0.4	2.6	0.5	2.7	1.341

N refers to the number of sickness benefit spells in the year preceding the intervention. The baseline variables are either measured in the calendar year preceding the sickness benefit spell or in the beginning (January 1st) of the same calendar year as the first day of the sickness benefit spell. a: χ^2^-test. b: t-test

^a^A Standardized mean difference equal to or higher than 1.96 indicates statistical significance at ≤0.05-level

Separate comparisons of baseline variables between each intervention municipality and its two corresponding control municipalities yielded similar results (not shown), except for Hjørring, which differed from its two control municipalities especially on gender, marital status, age and education.

The estimated HR for sickness benefit duration and duration until self-support is presented in Table 4 for all intervention and control municipalities jointly (row 1) and for each intervention municipality and its two corresponding control municipalities separately (rows 2–7). In the Table an HR >1 indicates a positive intervention effect. The intervention had on average a small positive but insignificant effect on the sickness benefit duration (HR 1.02, CI 0.97–1.07). Nor did the intervention have an effect on spells exceeding 52 weeks (HR 1.00, CI 0.84–1.19).

**Table 4:** Effects of the intervention on sickness benefit duration and duration until self-support

	Sickness benefit				Self-support				
	Effects for all spells		Distinct effects for spells exceeding 52 weeks		Effects for all spells		Distinct effects for spells exceeding 52 weeks		Number of sickness benefit spells in the intervention and control municipalities
	HR^b^	95% CI	HR^b^	95% CI	HR^b^	95% CI	HR^b^	95% CI	
All munici-palities^a^	1.02	0.97–1.07	1.00	0.84–1.19	0.99	0.96–1.02	1.03	0.94–1.12	282,103
Assens	**1.13** **(** ***p*** **< 0.001)**	1.08–1.18	1.12	0.95–1.31	**1.11** **(** ***p*** **< 0.001)**	1.06–1.17	1.16	0.99–1.36	33,361
Herning	**0.96** **(** ***p*** **= 0.039)**	0.93–1.00	**0.68** **(** ***p*** **< 0.001)**	0.60–0.76	1.00	0.97–1.04	0.99	0.88–1.12	52,359
Hjørring	0.97	0.89–1.06	1.30	0.93–1.82	**0.91** **(** ***p*** **= 0.041)**	0.82–1.00	1.02	0.76–1.37	26,382
Holbæk	**1.04** **(** ***p*** **= 0.040)**	1.00–1.09	1.06	0.91–1.23	1.01	0.97–1.06	0.97	0.84–1.13	37,803
Horsens	**1.07** **(** ***p*** **< 0.001)**	1.03–1.10	**1.36** **(** ***p*** **< 0.001)**	1.22–1.52	0.99	0.95–1.02	1.08	0.96–1.21	64,238
Kolding	1.00	0.97–1.03	1.05	0.93–1.17	0.97	0.94–1.01	0.97	0.86–1.09	67,960

Estimates are based on Cox proportional hazard rate models with baseline variables: gender, age, citizenship, months of schooling, marital status, pre-school children, previous sick-listing, number and length of hospitalizations, number of contacts with general practitioners (GPs), medical specialists, physiotherapists and psychologists. All baseline variables are time-invariant

^a^Standard errors are clustered by municipality

^b^An HR > 1 indicates a positive intervention effect. Statistically significant coefficients are in **bold** with *p*-value in ()

The insignificant overall effect on the sickness benefit duration masks great variation among the six intervention municipalities. The intervention had a positive and statistically significant effect on the sickness benefit duration in Assens, Holbæk and Horsens. With a hazard ratio of 1.13 (CI 1.08, 1.18) the effect was largest in Assens. The effect was insignificant in Hjørring and Kolding and negative and slightly statistically significant in Herning with a hazard ratio of 0.96 (CI 0.93–1.00). Generally, the effect sizes tended to increase when spells exceeded 52 weeks. However, as the CIs also broadened, the effect was positive and statistically significant only in Horsens (HR 1.36, CI 1.22–1.52), indicating that the positive intervention effect there was at least partly caused by a positive effect for spells exceeding 52 weeks. In contrast, the effect for spells exceeding 52 weeks was statistically significant and negative in Herning (HR 0.68, CI 0.60–0.76).

Table 4 also shows that the intervention had no overall effect on time to self-support (HR 0.99, CI 0.96–1.02) or on time to self-support for spells exceeding 52 weeks (HR 1.03, CI 0.94–1.12). The effect on time to self-support varied across municipalities. The intervention had a statistically significant and positive effect in Assens (HR 1.11, CI 1.06–1.17), a statistically significant and negative effect in Hjørring (HR 0.91, CI 0.82–1.00), and no effect in the other four municipalities. We found no significant effects on time to self-support for spells exceeding 52 weeks in any of the municipalities.

### Robustness analyses

In the first robustness analysis, we tested whether the pre-intervention development in the sickness benefit duration and time to self-support, respectively, differed significantly between the intervention municipalities and the control municipalities jointly as well as between each intervention municipality and its two corresponding control municipalities separately (see Additional file 2: Fig. S1 and Additional file 3: Fig. S2). The statistical analysis showed no statistically significant differences for both outcomes.^4^

In the second robustness analysis, we excluded the control municipality that was most deviant from each intervention municipality (Table 5). This did not notably change the magnitude of the estimated HRs for the joint analysis. However, this meant that the statistically significant result in Table 4 for Herning and Holbæk, respectively, for the sickness benefit duration (all spells) changed to become insignificant. For Hjørring the time to self-support (all spells) became insignificant as well. In contrast, the insignificant result for Hjørring for sickness benefit spells exceeding 52 weeks became statistically significant. All of the statistically significant results for Assens and Horsens in Table 4, however, showed to be robust.

**Table 5:** Effects of the intervention with one control municipality for each intervention municipality

	Sickness benefit				Self-support				
	Effects for all spells		Distinct effects for spells exceeding 52 weeks		Effects for all spells		Distinct effects for spells exceeding 52 weeks		Number of sickness benefit spells in the intervention and control municipalities
	HR^b^	95% CI	HR^b^	95% CI	HR^b^	95% CI	HR^b^	95% CI	
All municipalities^a^	1.03	0.97–1.09	0.99	0.84–1.17	1.00	0.97–1.03	0.98	0.90–1.07	201,583
Assens	**1.09** **(** ***p*** **= 0.001)**	1.03–1.15	0.98	0.81–1.18	**1.08** **(** ***p*** **= 0.006)**	1.02–1.15	1.11	0.92–1.34	23,389
Herning	1.00	0.96–1.04	**0.75** **(** ***p*** **< 0.001)**	0.65–0.86	1.02	0.97–1.06	0.93	0.80–1.07	40,301
Hjørring	1.04	0.94–1.14	1.26	0.89–1.76	0.96	0.87–1.06	0.97	0.71–1.33	13,284
Holbæk	1.02	0.97–1.07	**1.30** **(** ***p*** **= 0.006)**	1.08–1.56	0.98	0.93–1.04	0.90	0.75–1.07	30,197
Horsens	**1.11** **(** ***p*** **< 0.001)**	1.07–1.16	**1.31** **(** ***p*** **< 0.001)**	1.13–1.52	0.98	0.94–1.03	0.99	0.85–1.16	38,107
Kolding	0.99	0.96–1.03	0.97	0.86–1.10	0.99	0.96–1.03	1.02	0.90–1.15	56,305

Estimates are based on Cox proportional hazard rate models with baseline variables: gender, age, citizenship, months of schooling, marital status, pre-school children, previous sick-listing, number and length of hospitalizations, and number of contacts with general practitioners (GPs), medical specialists, physiotherapists and psychologists. All baseline variables are time-invariant

^a^Standard errors are clustered by municipality

^b^An HR > 1 indicates a positive intervention effect. Statistically significant coefficients are in **bold** with *p*-value in ()

In the third robustness analysis, we applied a Bonferroni-correction to each of the six separate municipal analyses. This adjustment yielded the same results as the second robustness analysis: the intervention effect for Herning and Holbæk for the sickness benefit duration (all spells) was no longer statistically significant and the intervention effect for Hjørring for the time to self-support (all spells) was no longer statistically significant either. Again, the statistically significant results for Assens and Horsens were robust.

In the fourth robustness analysis, we tested whether the participation of three of the control municipalities in ‘the Danish RTW programme’ affected the estimated intervention effects. One of Horsens’ two control municipalities participated in the programme. This was the municipality we removed in the second robustness analysis (Table 5), and removing it did not affect the magnitude of the intervention effect in Horsens. Both of Assens’ control municipalities participated in ‘the Danish RTW programme’. Therefore, we replaced the two control municipalities with the third best matching control municipality (Faaborg-Midtfyn). This did not change the estimated effect of the intervention on the sickness benefit duration (from HR 1.13, CI 1.08–1.18 to HR 1.10, CI 1.05–1.15). In contrast, the effect on time to self-support decreased significantly from a HR of 1.11 (CI 1.06–1.17) to 1.02 (CI 0.97–1.07). In conclusion, while the three control municipalities’ participation in ‘the Danish RTW programme’ apparently did not affect the estimated intervention effect on the sickness benefit duration, it might have led to an overestimation of the estimated intervention effect on time to self-support.

## Discussion

Analysing the six intervention municipalities jointly, we found that the intervention had no overall effect on the sickness benefit duration or on time to self-support. The separate analyses of the intervention municipalities showed, however, notable differences in the effect of the intervention among the six municipalities. On the one hand, it could be argued that there will often be differences when testing multiple models, i.e. that by chance some will be statistically significant while others will not. On the other hand, after applying a Bonferroni-correction some of the effects of the separate analyses of the intervention municipalities remained statistically significant, suggesting that these effects were not found by chance. Variation in the municipalities’ implementation of the intervention could potentially explain the differences in the effect across the six municipalities. Previous RTW-interventions have shown that implementation often varies and thereby may influence to what extent the intervention can lead to the expected effects [25–28, 33, 35]. The degree of successful implementation of the intervention might, therefore, account for some of the variation between municipalities.

In the context of this study, we can provide only a crude analysis of these associations. We find some association between implementation and effects, e.g. positive intervention effects on the sickness benefit duration in the two municipalities (Assens and Horsens) that implemented all or almost all of the intervention elements, and a negative effect in the one municipality (Herning) with the lowest number of implemented intervention elements (Tables 1 and 4). However, in Hjørring, we found no association between implementation and effects: despite the implementation of almost all of the intervention elements, we found no effect on the sickness benefit duration and a negative effect on time to self-support. These findings suggest that based on this crude assessment the extent to which the elements have been implemented can explain only some of the variation. Therefore other aspects should be considered also.

The complexity of the intervention might also have made it difficult to acieve positive results [22–24]. Furthermore, while the intervention focussed on a variety of measures to improve the quality and efficiency of sickness benefit case management, it may have had too little focus on case management elements that researchers have shown to be important for RTW, e.g. involvement of workplaces [7, 40, 41] and the quality of the SIO’s communication with the sick-listed [42]. Thus, the involvement of workplaces through increased use of company consultants was only one of many elements of the intervention and SIO communication was not specifically addressed.

Changes in the control group constitute yet another challenge for effect evaluations of intervention studies. In our study, information from the 12 control municipalities suggests that they continuously developed their OSBM policies to varying degrees during the study period. These developments may have enhanced the control municipalities’ OSBM differently, causing some of the variation. Similarly, if the widespread development of OSBM in the control municipalities significantly reduced the sickness benefit duration, this reduction would reduce the magnitude of the intervention effect.

Three of the 12 control municipalities participated in ‘the Danish RTW programme’. However, our robustness analysis showed that this participation did not lead to an underestimation of our estimated effects.

In one of the six intervention municipalities, Hjørring, the distribution of several baseline variables was substantially different from that of its two control municipalities. However, these differences do not lead us to reservations about the comparability. We selected control municipalities with similar ‘structural conditions’ and geographic location to those in the intervention municipalities to ensure that the intervention and control municipalities had characteristics generating a population of sickness beneficiaries with the same average number of sickness benefit weeks. Consequently, we may assume that the sum of the characteristics of individuals in the intervention and control municipalities generated the same expected subsequent sickness benefit duration (disregarding a possible intervention effect). However, this result does not require the distribution of each characteristic in the intervention and control municipalities to be the same.

### Strengths and limitations of the study

One strength of our study is the use of individual-level administrative register data from several national registers, enabling us to include all sickness benefit spells exceeding four weeks in the intervention and control municipalities within the study period. Thus, the study is fully representative of the target population, increasing external validity. With data on more than 280,000 spells, this study had sufficient power to detect even small effects. Using register data, we also avoided attrition and recall bias, thereby increasing the study’s internal validity.

A second strength is the study design. In contrast to most non-randomised studies, we had access to data allowing us to select control municipalities that resembled the intervention municipalities concerning both structural conditions and geographic location affecting the population of sickness beneficiaries. Consequently, our selection procedure enhanced the comparability of the intervention and control municipalities.

A third strength is the collection of information about the OSBM in the control municipalities during the study period. This information allowed us to conduct a robustness analysis where we excluded control municipalities with interventions most similar to the intervention tested here.

The major limitation of the study is that it was not planned or executed as an RCT. With this constraint we had to apply a quasi-experimental (DID) design and, therefore, cannot completely eliminate the possibility that the non-randomisation yielded bias in the estimated intervention effects. Another limitation is that the administrative registers do not include information on the sick leave diagnoses. With such information we could have studied whether the intervention had an effect for beneficiaries with particular diagnoses.

## Conclusions

Overall, this study does not support the hypothesis that an intervention focussing on improving the quality and efficiency of sickness benefit case management reduces the sickness benefit duration and time to self-support compared to ordinary sickness benefit management. Nonetheless, the study shows that the magnitude of the intervention effect varied among the six intervention municipalities. While differences in implementation appear to play a role, they cannot explain all variance. Other factors, such as the complexity of the intervention, might also have played a role. As we used a non-randomised DID design, further research on the effects of interventions for sickness absence beneficiaries would benefit from an RCT design. However, while this might reduce the risk of bias in the estimated intervention effects a comprehensive process evaluation is also needed to better understand the role of implementation in complex interventions.

## Additional files

Additional file 1: Table S1. Predicted average sickness benefit rates in the intervention and the control municipalities. The data stems from the report ‘Framework conditions of Danish municipalities in relation to employment interventions’ [30]. The data consist of a broad range of unique individual, municipal and regional level administrative register data for all Danish municipalities. Graversen et al. [30] coded 145 variables for measuring several individual, municipal and regional characteristics that may affect the number of persons receiving sickness benefits exceeding four weeks. The individual characteristics included labour market experience, housing composition, and use of health care services. The municipal and regional characteristics included unemployment rate in the commuting area, number of inhabitants, and composition of job skills among employed people. (DOCX 14 kb)

Additional file 2: Fig. S1. Pre-intervention development in hazard ratio (HR) of sickness benefit duration. Intervention and control municipalities separately. The data stem from the present study and the graphs are, thus, based on individual level administrative register data from a selection of Danish municipalities. The graphs show whether the pre-intervention development in the sickness benefit duration differed significantly between the intervention municipalities and the control municipalities jointly (panel a) as well as between each intervention municipality and its two corresponding control municipalities separately (panel b-g). (DOCX 351 kb)

Additional file 3: Fig. S2. Pre-intervention development in hazard ratio (HR) of time to self-support. Intervention and control municipalities separately. The data stem from the present study and the graphs are, thus, based on individual level administrative register data from a election of Danish municipalities. The graphs show whether the pre-intervention development in time to self-support differed significantly between the intervention municipalities and the control municipalities jointly (panel a) as well as between each intervention municipality and its two corresponding control municipalities separately (panel b-g). (DOCX 304 kb)

## Abbreviations

**CI**: Confidence intervals
**DID**: Difference-in-difference
**DREAM**: ‘Danish Register for Evaluation of Marginalisation’
**Falck**: Falck Health Care
**GP**: General practitioner
**HR**: Hazard ratio
**OSBM**: Ordinary sickness benefit management
**RCT**: Randomised controlled trial
**RTW**: Return-to-work
**SE**: Standard error
**SIO**: Social insurance officer
**TVC**: Time varying covariate
**VCE**: Stata’s robust-clustered SE option

## Footnotes

1 In some cases a person had two sickness benefit spells interrupted by a few weeks without benefits, e.g. because of holidays. To avoid such misclassifications, we coded two spells separated by a period of under five weeks without sickness benefit as one consecutive spell

2 In both the intervention and control municipalities almost 12% of the spells had a duration exceeding 52 weeks

3 As the clustered (robust) SEs may also be biased with few clusters [35:313], we report the conventional (unclustered) SEs for the intervention effects of the separate analyses of each of the six intervention municipalities. As a robustness analysis we performed an analysis with only one control municipality for each of the six intervention municipalities, i.e. a cluster-specific fixed-effects model [43]

4 2nd year before the initiation of the intervention is reference year in both of the figures and the robustness test.
