# Supplementary material for: Improving the effectiveness of sickness benefit case management through a public-private partnership? A difference-in-difference analysis in eighteen Danish municipalities
Source: BMC Public Health. 2017 Apr 18;17:329. doi: 10.1186/s12889-017-4236-5 (PMC5395754; doi:10.1186/s12889-017-4236-5)
Supplement: Supplementary file 1 — Predicted average sickness benefit rates in the intervention and the control municipalities. The data stems from the report ‘Framework conditions of Danish municipalities in relation to employment interventions’ [30]. The data consist of a broad range of unique individual, municipal and regional level administrative register data for all Danish municipalities. Graversen et al. [30] coded 145 variables for measuring several individual, municipal and regional characteristics that may affect the number of persons receiving sickness benefits exceeding four weeks. The individual characteristics included labour market experience, housing composition, and use of health care services. The municipal and regional characteristics included unemployment rate in the commuting area, number of inhabitants, and composition of job skills among employed people. (DOCX 14 kb) [file 12889_2017_4236_MOESM1_ESM.docx]

Appendix 1

**Table A1** Predicted average sickness benefit rates in the intervention and the control municipalities

| Intervention municipalities | Predicted sickness benefit weeks | Control municipalities | Predicted sickness benefit weeks |
| --- | --- | --- | --- |
| Assens | 2.7 | Nordfyns | 2.8 |
|  |  | Nyborg | 2.6 |
| Herning | 2.5 | Ringkøbing-Skjern | 2.6 |
|  |  | Ikast-Brande | 2.4 |
| Hjørring | 2.6 | Jammerbugt | 2.7 |
|  |  | Brønderslev | 2.6 |
| Holbæk | 2.5 | Sorø | 2.4 |
|  |  | Lejre | 2.3 |
| Horsens | 2.5 | Favrskov | 2.4 |
|  |  | Silkeborg | 2.4 |
| Kolding | 2.4 | Middelfart | 2.5 |
|  |  | Vejle | 2.3 |

Source: Graversen et al. [31].
