# Supplementary material for: Improving the effectiveness of sickness benefit case management through a public-private partnership? A difference-in-difference analysis in eighteen Danish municipalities
Source: BMC Public Health. 2017 Apr 18;17:329. doi: 10.1186/s12889-017-4236-5 (PMC5395754; doi:10.1186/s12889-017-4236-5)
Supplement: Supplementary file 2 — Pre-intervention development in hazard ratio (HR) of sickness benefit duration. Intervention and control municipalities separately. The data stem from the present study and the graphs are, thus, based on individual level administrative register data from a selection of Danish municipalities. The graphs show whether the pre-intervention development in the sickness benefit duration differed significantly between the intervention municipalities and the control municipalities jointly (panel a) as well as between each intervention municipality and its two corresponding control municipalities separately (panel b-g). (DOCX 351 kb) [file 12889_2017_4236_MOESM2_ESM.docx]

Appendix

Figure A1 panel a

Figure A1 panel b

Figure A1 panel c

Figure A1 panel d

Figure A1 panel e

Figure A1 panel f

Figure A1 panel g
