# Supplementary material for: Improving the effectiveness of sickness benefit case management through a public-private partnership? A difference-in-difference analysis in eighteen Danish municipalities
Source: BMC Public Health. 2017 Apr 18;17:329. doi: 10.1186/s12889-017-4236-5 (PMC5395754; doi:10.1186/s12889-017-4236-5)
Supplement: Supplementary file 3 — Pre-intervention development in hazard ratio (HR) of time to self-support. Intervention and control municipalities separately. The data stem from the present study and the graphs are, thus, based on individual level administrative register data from a election of Danish municipalities. The graphs show whether the pre-intervention development in time to self-support differed significantly between the intervention municipalities and the control municipalities jointly (panel a) as well as between each intervention municipality and its two corresponding control municipalities separately (panel b-g). (DOCX 304 kb) [file 12889_2017_4236_MOESM3_ESM.docx]

Appendix

Figure A2 panel a

Figure A2 panel b

Figure A2 panel c

Figure A2 panel d

Figure A2 panel e

Figure A2 panel f

Figure A2 panel g
